# Identification of single nucleotide polymorphisms in the bovine Toll-like receptor 1 gene and association with health traits in cattle

**DOI:** 10.1186/1297-9716-43-17

**Published:** 2012-03-14

**Authors:** Christopher D Russell, Stephanie Widdison, James A Leigh, Tracey J Coffey

**Affiliations:** 1 Bovine Genomics Group, Institute for Animal Health, Compton, Berkshire, RG20 7NN, UK; 2 The School of Veterinary Medicine and Science, The University of Nottingham, Sutton Bonington Campus, Sutton Bonington, Leicestershire, LE12 5RD, UK

## Abstract

Bovine mastitis remains the most common and costly disease of dairy cattle worldwide. A complementary control measure to herd hygiene and vaccine development would be to selectively breed cattle with greater resistance to mammary infection. Toll-like receptor 1 (TLR1) has an integral role for the initiation and regulation of the immune response to microbial pathogens, and has been linked to numerous inflammatory diseases. The objective of this study was to investigate whether single nucleotide polymorphisms (SNPs) within the bovine TLR1 gene (bo*TLR1*) are associated with clinical mastitis (CM).

Selected bo*TLR1 *SNPs were analysed within a Holstein Friesian herd. Significant associations were found for the tagging SNP -79 T > G and the 3'UTR SNP +2463 C > T. We observed favourable linkage of reduced CM with increased milk fat and protein, indicating selection for these markers would not be detrimental to milk quality. Furthermore, we present evidence that some of these bo*TLR1 *SNPs underpin functional variation in bovine TLR1. Animals with the GG genotype (from the tag SNP -79 T > G) had significantly lower bo*TLR1 *expression in milk somatic cells when compared with TT or TG animals. In addition, stimulation of leucocytes from GG animals with the TLR1-ligand Pam3csk4 resulted in significantly lower levels of CXCL8 mRNA and protein.

SNPs in bo*TLR1 *were significantly associated with CM. In addition we have identified a bovine population with impaired bo*TLR1 *expression and function. This may have additional implications for animal health and warrants further investigation to determine the suitability of identified SNPs as markers for disease susceptibility.

## Introduction

Bovine mastitis is an inflammatory udder disease of great economic importance to the dairy industry. Mastitis is caused mainly by intramammary bacterial infection hence effective disease control measures rely upon farm management practices to limit the duration of infection and to restrict contagious spread of pathogens through the herd. As a result of these control measures opportunistic pathogens such as *Escherichia coli *and *Streptococcus uberis *predominate, and are now the leading cause of mastitis in the UK and common causes of mastitis worldwide [1]. Vaccination programmes may be required to further control mastitis [2], however no effective vaccines are currently available. An additional long term and complementary strategy is the genetic selection of cattle that are less prone to mastitis. A lack of phenotypic data on clinical mastitis (CM) limits many breeding programmes, which select on the basis of a lowered somatic cell count (SCC) as a surrogate of CM [3]. The correlation between basal SCC and CM continues to be questioned [4,5] and the elucidation of more accurate genetic risk factors for CM is required. The identification of single nucleotide polymorphisms (SNPs) within genes involved with the mammary innate immune response are receiving particular interest as DNA markers for CM susceptibility [6-9].

The innate immune system provides an early defence to microbial pathogens [10] and involves cell surface proteins from the Toll-like receptor (TLR) family. TLRs are a structurally conserved type I membrane-bound class of pathogen recognition receptor (PRR), that can differentiate between an array of pathogen-associated molecular patterns (PAMPs) [11,12]. Functional expression of TLRs on resident mammary immune cells and epithelia has been shown to be important in the initiation and control of innate immune responses towards mastitis-causing bacteria [13-16]. Ten functional bovine (bo) TLRs have been characterised (Coffey et al. unpublished) [17], revealing close identity to their human orthologues [18]. Each individual TLR possesses its own ligand recognition repertoire, and upon stimulation activate internal adaptor proteins to initiate signalling for the NFκB-mediated production of pro-inflammatory cytokines including the chemokine CXCL8 [19]. During mastitis, CXCL8 production increases rapidly [20] providing a potent chemotactic gradient to promote neutrophil movement into the mammary gland, a vital early defence mechanism against infection.

The presence of SNPs within mammalian *TLR*s has been shown to predispose susceptibility to a number of inflammatory diseases [21-23]. TLR1 variants have been postulated to underlie the altered immune responses to a wide spectrum of bacterial pathogens. In humans, a non-synonymous *TLR1 *SNP located within the transmembrane domain, which had an impact on TLR1 trafficking, NFκB activity and cytokine output, was found to have strong associations with susceptibility to and progression of infection by Gram positive bacteria and *Mycobacterium leprae *[24,25]. Polymorphisms affecting bo*TLR1 *may therefore predispose excessive or poor immune responses to pathogens and hence could confer an increased risk of mammary infection.

Mammalian TLR1 is a member of a unique TLR sub-family that includes TLR6 and TLR10. All have the ability to form heterodimers with TLR2 to expand the repertoire of recognised ligands. TLR1/TLR2 complexes mediate cellular responses to natural triacylated lipoprotein structures [26,27], which are cell wall constituents of Gram-positive and Gram-negative bacteria [28]. TLR6/TLR2 complexes expand ligand recognition to diacylated lipoproteins, although in cattle the boTLR1/TLR2 complex may be further activated by diacylated lipoproteins [29]. Whilst more investigation is required to uncover their function, TLR10/TLR2 complexes may extend recognition of TLR1 agonists via an unknown signalling mechanism [30]. Phylogenetic analysis of bo*TLR1, 6 *and *10 *reveals common ancestry with all three genes located in tandem to form a ~69 kb *TLR6-TLR1-TLR10 *gene cluster on *Bos taurus *chromosome 6 (bta6) [31]. Genome-wide quantitative trait locus (QTL) studies reveal the bo*TLR6-TLR1-TLR10 *gene cluster to be situated within a dense QTL region for a number of milk production traits and CM [32,33]. Furthermore, bo*TLR1 *has been highlighted as a strong candidate for underlying QTL regions for disease resistance such as mastitis [34].

Despite strong QTL evidence, associative studies between bo*TLR1 *and mastitis are limited. The bo*TLR1 *coding sequence (CDS) is highly polymorphic, containing many SNPs across several cattle breeds [35]. Four SNPs within the CDS of bo*TLR1 *were identified in Holstein cows [36], but only one non-synonymous SNP, located within the transmembrane domain, was reported to result in highly significant differences in SCC between all genotype populations. More investigation is required to verify such studies, and to establish associations with novel bo*TLR1 *SNPs/haplotypes. More importantly, the ability to use data indicating actual CM incidence, rather than a surrogate such as SCC, and associate this with specific genetic markers has the potential to yield more accurate information.

We report the identification of bo*TLR1 *SNPs within coding and non-coding regions, and their potential as genetic markers for mastitis. We further demonstrate evidence of variable receptor response between defined bo*TLR1 *SNP genotypes.

## Materials and methods

### Ethics statement

All experiments conformed to local and national guidelines on the use of experimental animals.

### Sample population

The herd of Holstein Friesian cows used during this project (Mayfield Dairy, Institute for Animal Health, Compton, UK) contributes to the National Milk Records (NMR) [37] which submits production records for each animal to a database accessed through the InterHerd software programme (University of Reading/PAN Livestock services team, UK). This, along with health data held locally, provided comprehensive information for each cow within the herd. All animal data were recorded within the years 2001-2010, during which, the mean CM incidence for the milking herd was 60 cases per 100 cows per year.

### Isolation and extraction of genomic DNA from milk somatic cells

Milk samples (50 mL) were held on ice prior to centrifugation (3000 *g*; 5 min; 4°C). Supernatants were carefully removed along with any residual fat layer. Remaining casein micelles were removed using an adaptation of a procedure devised previously [38]. In brief, cell pellets were re-suspended in 1.5 mL PBS/EDTA solution (1 mL PBS, 300 μL 0.5 M EDTA and 200 μL TE buffer - 10 mM-Tris-HCl-1 EDTA pH 7.6), mixed well and allowed to stand at room temperature (RT) for 10 min. Cells were re-pelleted by centrifugation (7000 *g*; 1 min; RT), the supernatant discarded and cells re-suspended in 1.5 mL of TE buffer. Cells were pelleted again (7000 *g*; 1 min) and resuspended in 200 μL PBS.

Milk somatic cells were processed using the Qiagen DNeasy blood and tissue kit for genomic DNA isolation (Qiagen Ltd, Sussex, UK). Briefly, 20 μL proteinase K solution (Qiagen Ltd.) was added to resuspended cells and mixed well before being incubated overnight at 4°C. To ensure RNA-free genomic DNA, 4 μL RNase A (100 mg/mL) was added following overnight incubation and samples left for 2 min at RT. DNA was extracted using the column protocol according to manufacturer's instructions (Qiagen Ltd). The quantity and quality of DNA was analysed using a NanoDrop 8000 spectrophotometer (Thermo Scientific, USA) and agarose gel electrophoresis.

### Identification and sequencing of sample population for bo*TLR1 *SNPs

Bo*TLR1-*specific primers for PCR amplification of coding and non-coding genomic regions were designed based on the *Bos taurus *genome annotation build: Btau_4.0 (NC_007304.4). Sequencing of PCR amplicons from a DNA pool comprising animals with high (greater than six lifetime CM cases) or no mastitis case histories was initially chosen as a method of accelerating detection of disease-associated SNPs. Selected animals were unrelated and shared similar ages and lactation histories. PCR was performed using DreamTaq™ DNA polymerase (Fermentas UK, York, UK) following the manufacturer's protocol. Sequencing of purified PCR products was carried out using BigDye Terminator v3.1 Cycle Sequencing Kit (Applied Biosystems, Warrington, UK). Samples were sequenced by capillary electrophoresis on an ABI PRISM 310 Genetic Analyser (Applied Biosystems), and analysed using the Sequencher™package version 4.1.4 (Gene Codes Corporation, Ann Arbor, MI, USA). All primers (Additional file 1) were designed using Primer3 software [39] and synthesised by Sigma-Genosys Ltd. (Haverhill, UK). All bo*TLR1 *SNP offsets are given relative to their position (bp) from the A nucleotide of the ATG codon. Sequencing of PCR amplicons was extensively used to genotype the herd population.

### Restriction fragment length polymorphism (RFLP) - PCR analysis

In addition to sequencing of PCR amplicons, SNPs +798 C > T and +1762A > G were genotyped using RFLP analysis of PCR products; whereby restriction enzyme MboII cuts in the presence of the C allele for SNP +798 C > T, and restriction enzyme BclI cuts only in the presence of the A allele (Isoleucine) for the non-synonymous SNP +1762A > G; as described previously [36]. Restriction digests were performed in a final volume of 20 μL containing 10 μL PCR product and 2U of appropriate restriction enzyme. Digests were incubated at the recommended temperature for 2 h, with a subsequent heat inactivation of enzyme at 65°C for 20 min. Homozygous and heterozygous samples, confirmed by sequencing, were included as controls.

### Identifying associations between SNPs and clinical mastitis and milk quality

Identified SNPs were correlated to health or milk productivity trait data available through the InterHerd software programme and NMR data. These included CM case histories, milk SCC, mean 305 d milk yields and mean milk protein/fat concentrations. All Animal data were recorded within and up to, the first three lactations only. Median SCC was transformed logarithmically as NEWSCC = LOG2 (SCC/100 000) + 3. This model accounts for the skewness of data distribution as used previously [3]. CM was analysed for presence or absence of cases across the entire milking tenure of their first three lactations. Mean incidence of CM is presented as cases per cow per year ([total number of cases/total number of milking days]*365) within the first three lactations. CM was defined as an individual clinically apparent event, coupled with a rise in milk SCC and/or isolation of pathogen, recorded on a single day within any/all quarters during a lactation period. Repeated cases within the same quarter were noted but only regarded as a new case after seven days return to milking.

### Statistical analysis

Genotypic frequencies were tested for deviation from Hardy-Weinberg equilibrium according to Chi^2 test and level of significance at 1 degree of freedom. Haplotypes and linkage disequilibrium (LD) scores (based on the r^2 ^statistic), were analysed using the Haploview programme [40]. Owing to substantial positive skewness (due to zero values) of data distribution, mean CM rate was transformed: NEWCM = LOG10 (CM + 1). A genotypic model was used for separate genotype pair-wise comparisons. One-way analysis of variance (ANOVA) and Tukey's multiple comparisons for CM rate, milking traits and functional data means, were performed using Prism^® ^5.04 software (Graph Pad Software, Inc, CA, USA). Presence of any CM event across all lactations (CM recorded = 1, no recorded CM = 0) between genotypes were pair-wise tested for significance by a Chi^2 odds ratio test using Minitab^® ^16 software (Minitab Statistical Software, Inc, PA, USA). Calculation of Pearson correlation coefficients between factors and analysis of CM response in a General linear model (GLM) was performed using Minitab^® ^16 software.

### Isolation of polymorphonuclear leukocytes from bovine blood

For stimulation assays, polymorphonuclear leukocytes (PMNs) were isolated from 50 mL blood samples of healthy, age-matched, genotyped cattle that had recorded no intramammary infection in the two weeks prior to sample collection. Briefly, blood was collected into EDTA (final concentration of ~1.5%w/v), centrifuged at RT (15 min; 1000 *g*) and the plasma and buffy coat phases carefully removed. For every 2.5 g of lower blood phase, 10 mL of H_2_O was added. The mixture was agitated and left for 40 s before adding half the water volume of NaCl/PO_4 _reagent (2.7% NaCl/PO_4 _reagent: 2.7 g NaCl into 10 mL 0.132 M phosphate buffer (18.74 g Na_2_HPO_4 _and 17.96 g KH2PO_4 _into 1 L H_2_O, pH 6.8) and made up to 100 mL with H2O) to restore the isotonic strength. PMNs were pelleted by centrifugation (125 *g*; 10 min; RT), the supernatant discarded and the cells re-suspended in 1.5 mL of PBS. This was repeated twice to wash the cells. Typically 1 × 10^8 ^PMN cells were obtained per 50 mL sample. FACS analysis of polymorphic PMN samples suggested little difference in cell populations and purity obtained from the different genotypes.

Isolated PMNs were seeded to 1 × 10^6^/1 mL in RPMI 1640 + GlutaMAX™with 10% foetal calf serum (FCS) (both Invitrogen Ltd. UK) and 100 units mL^-1^/100 μg mL^-1 ^Penicillin/Streptomycin (Sigma, St Louis, MO, USA). Prior to stimulation, seeded cells were incubated at 37°C, in 5% CO_2 _for approximately 18 h in 24 well tissue culture plates.

### Stimulation of PMNs with TLR ligands

All TLR ligands (InvivoGen, San Diego, CA, USA) were reconstituted in sterile endotoxinfree water according to the manufacturers' instructions, to a concentration of 1 mg mL^-1^, and stored at 20°C. The synthetic TLR1-specific ligand PAM3 was added to the cells in 100 μL of culture media, at final concentrations of 100 ng/mL, 250 ng/mL and 500 ng/mL. A media control (containing no ligand) was used. LPS (100 ng/mL) was used as a non-TLR1 stimulant. Stimulated cells (4 h and 8 h post stimulation) were lysed and total RNA extracted. Supernatants were taken 24 h post stimulation for detection of cytokine production by ELISA.

### RNA isolation and cDNA synthesis

Pelleted cell samples were lysed in RLT buffer for total RNA extraction using the RNeasy mini kit (Qiagen Ltd) according to manufacturer's instructions. Samples were DNase treated (DNAfree, Ambion, Austin, TX, USA) and analysed for quantity and quality. Total RNA was used for cDNA synthesis using SuperScript II Reverse Transcriptase (Invitrogen Ltd. UK) using the recommended protocol. Quantification of cDNA was carried out using the NanoDrop 8000 spectrophotometer.

### Q-PCR analysis

Quantitative PCR (Q-PCR) was performed with the ABI Prism 7500 FAST Sequence Detection System (Applied Biosystems) using FAST Universal Mastermix (Applied Biosystems) according to the manufacturer's protocol. Taqman^® ^probe and primers for *GAPDH, CXCL8 *and *Bcl2A1 *have been designed previously [41-43]. Taqman^® ^probe and primers for bo*TLR1 *were designed from sequenced templates, avoiding any SNPs, using the Primer3 software [39] (Forward: 5'-GCA CCA CAG TGA GTC TGG AA -3', Reverse: 5'GTA CGC CAA ACC AAC TGG AG -3', Probe: 5'-TGT GTG CTT GAT GAT AAT GGG TGT CCT -3'). All primers and probes were synthesised by Sigma-Genosys Ltd. and Eurogentec Ltd. (Romsey, UK) respectively. Probes were labelled at the 5' end with the reporter dye FAM (6-carboxyfluorescein) and at the 3' end with the quencher dye TAMRA (6-carboxytetramethylrhodamine). 100 ng of cDNA template from each animal were tested in triplicate and quantified by comparison with a standard curve from plasmid DNA of known copy number. Relative target gene expression was then calculated by normalising to the mean expression levels of the housekeeping genes glyceraldehyde-3-phosphate dehydrogenase (*GAPDH*) and ribosomal protein, large, P2 (*RPLP2*). Analysis was performed using Microsoft^® ^Excel 2008 (Microsoft Co., Redmond, WA, USA) and Prism^® ^5.04 software. Differences between groups were assessed by one-way analysis of variance (ANOVA) and Tukey's multiple comparisons.

### ELISA

Levels of CXCL8 were determined by luminescent ELISA, using a pair of commercial human IL-8 specific antibodies (R&D Systems, Oxon, UK) according to a modified luminescence-based ELISA described previously [44]. Samples were tested in triplicate, with recombinant bovine CXCL8 (Kingfisher Biotech, Inc., MN, USA) as the standard. Levels of bovine IL-6 were determined using a commercial TMB ELISA kit according to the manufacturer's protocol (Thermo Fisher Scientific, Inc. USA). Differences between groups were assessed by one-way analysis of variance (ANOVA) and Tukey's multiple comparisons.

## Results

### Characterisation of bo*TLR1 *SNPs

**Figure 1:**
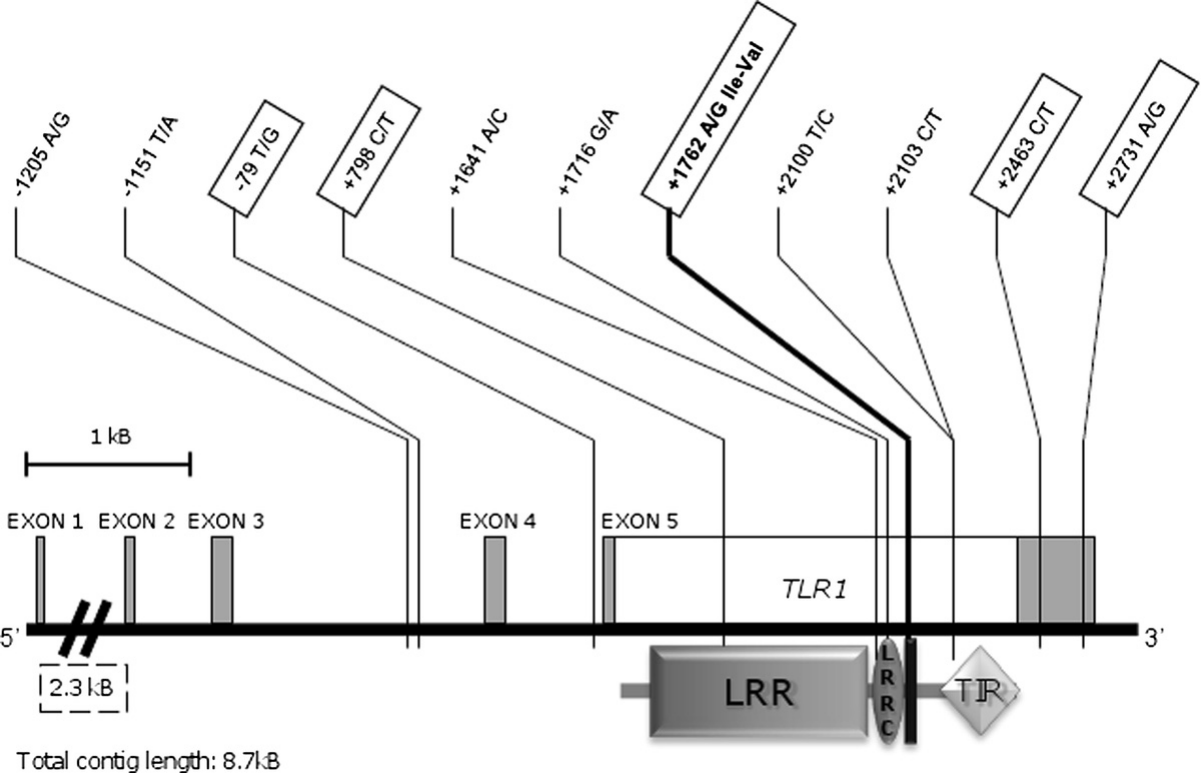
**Schematic diagram showing the positions of identified SNPs within the bovine *TLR1 *gene**. Open box represents coding exon (CDS = 2379 bp), grey boxes represent non-coding untranslated exonic regions (UTR). A TLR1 protein domain architecture diagram, designed using web-based programmes [47,48], is illustrated below the CDS. The non-synonymous +1762A > G SNP (in bold), is located within the transmembrane domain (vertical black bar). LRR - leucine-rich repeat domains (19 in total), LRR C -leucine-rich repeat C terminal domain, TIR - Toll-Interleukin 1-resistance domain. Sequence shown from Btau_4.0 sequence (NC_007304.4), bta6: 60355 k-60363 k. Boxed SNPs have been sequenced extensively for trait associations.

The genomic organisation of bo*TLR1 *was confirmed using internal cDNA sequencing, and analysis of EST data submitted to the NCBI database. As described previously [31], the full length bo*TLR1 *mRNA transcript comprises five exons and four introns. The CDS for full length boTLR1; a 2379 bp transcript corresponding to a 793aa chain [45], is located within exon 5. Sequencing of bo*TLR1 *within a Holstein Friesian herd (*n *= 246) revealed eleven SNPs; five of which are novel (Table 1). Eight of the eleven SNPs were exonic, six were located in the CDS, of which only one was a non-synonymous change (+1762A > G, Iso > Val) positioned within the transmembrane domain, while the remaining two were located in the non-coding 3'UTR. The three non-exonic SNPs were identified within the introns of the 5' UTR (Figure 1).

**Table 1:** Identified SNPs within bo*TLR1 *from Holstein Friesians.

SNP	Genomic (bta6) position	Amino acid position	Amino acid	Restriction site presence	Previously reported	dbSNP ID
-1205A > G	60440366	-	-	-	No	-

-1151T > A	60440312	-	-	-	No	-

-79T > G	60439240	-	-	-	No	-

+798 C > T	60438363	266	Phe-Phe	MboII	Yes ^1^	ss73689409

+1641A > C	60437520	547	Ser-Ser	Bst X-I	Yes^2^	ss73689413

+1716 G > A	60437445	572	Lys-Lys	Bsi Y	Yes^2^	ss73689414

+1762A > G	60437324	587	Ile-Val	Bcl I	Yes^2^	ss73689415

+2100 C > T	60436989	700	Phe-Phe	-	Yes^2^	ss73689417

+2103 T > C	60436986	701	Val-Val	BsmAI	Yes^2^	ss73689418

+2463 C > T	60436698	-	-	MsII	No	-

+2731A > G	60436430	-	-	-	No	-

Abbreviations: bta6 = *Bos taurus *chromosome 6, dbSNP = Single-Nucleotide Polymorphism Database [46]. All bo*TLR1 *sequences were aligned with the published Btau_4.0 sequence NC_007304.4 (underlined allele denotes the published sequence). Light shading - synonymous coding SNPs, dark shading -non-synonymous coding SNP. All bo*TLR1 *SNP offsets are given relative to their position (bp) from the A nucleotide of the first ATG codon. ^1 ^[31], ^2 ^[35].

### Genotypes and allelic frequencies

**Figure 2:**
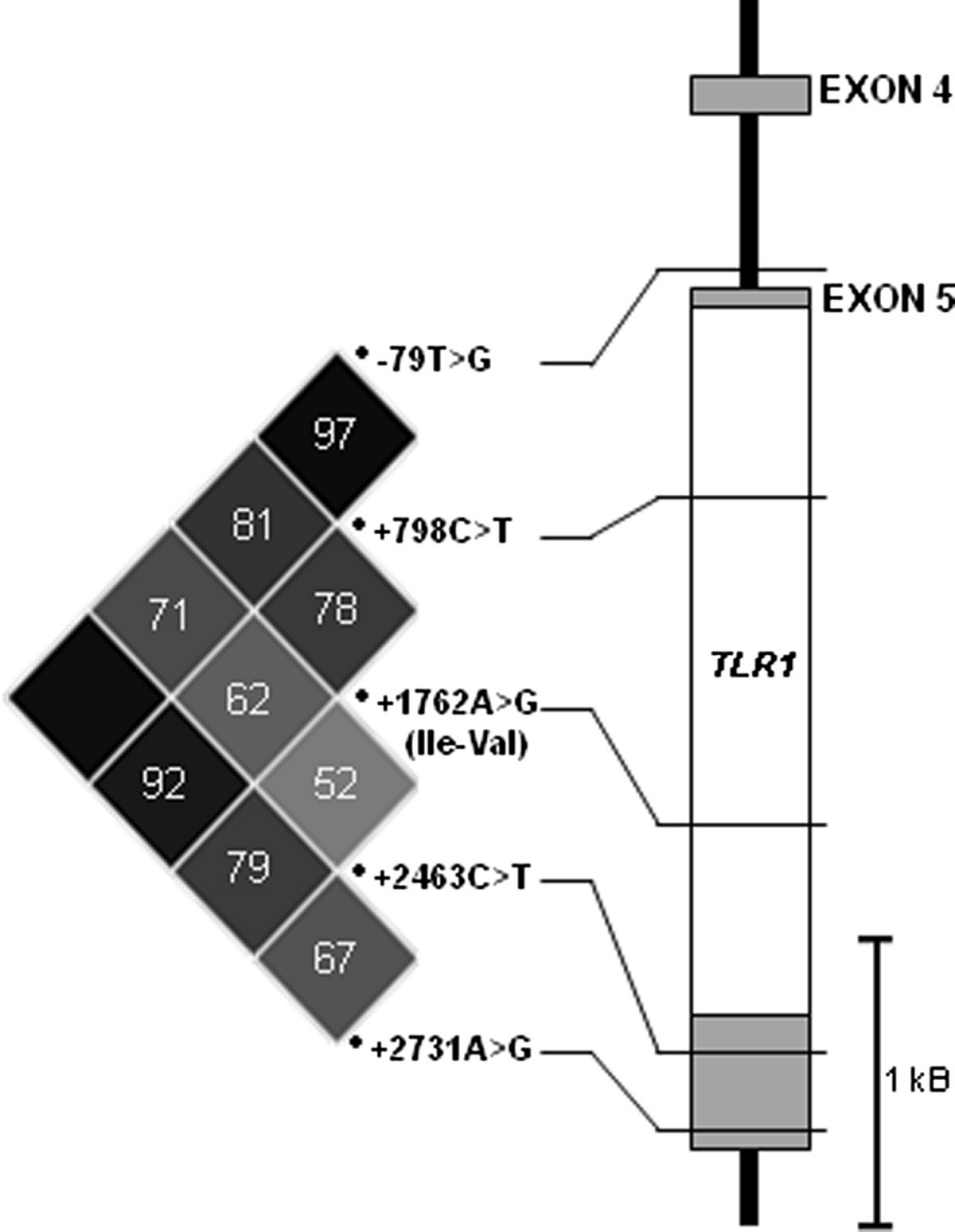
**Linkage disequilibrium (LD) plot for five extensively genotyped SNPs in relation to the bo*TLR1 *gene model**. Open box represents bo*TLR1 *coding exon, grey boxes represent non-coding untranslated (UTR) exonic regions. The non-synonymous amino acid change is shown in brackets below corresponding coding SNP. The graphical representation, generated by Haploview, of the r^2 ^LD relationship between each SNP is expressed on a gray-scale gradient; where black - very high LD (r^2 ^= 1.0) and white - very low LD (< 0.1). The r^2 ^value for each pair-wise comparison is shown, where no value is indicated r^2 ^= 1.

Five bo*TLR1 *SNPs: -79 T > G, +798 C > T, +1762A > G, +2463 C > T, and +2731A > G (Figure 1) were detected within the herd (*n *= 156, 246, 125, 225 and 232 respectively). The genotypic and allelic frequencies for each SNP are similar, with heterozygotes the most abundant genotypes (Table 2). All SNPs were found to be in agreement with Hardy-Weinberg equilibrium. The three SNPs -79 T > G, +798 C > T, and +2731A > G were in strong linkage disequilibrium (LD = > 0.9) (Figure 2) and as a result only the SNP -79 T > G was used for association analysis. LD between the tagging SNP -79 T > G and the non-synonymous +1762A > G was moderate (LD = ~0.8). LD between SNPs -79 T > G and +2463 C > T was lower. The lowest LD was observed between SNPs +1762A > G and +2463 C > T (Figure 2). Consequently, analysis of association with traits for SNPs -79 T > G, +1762A > G and +2463 C > T was performed individually. Preliminary haplotype construction for SNPs 79 T > G, +798 C > T, +1762A > G, +2463 C > T, and +2731A > G, yielded two major haplotypes of G_79_T_798_A_1762_C_2463_G_2731 _(frequency 0.42) and T_79_C_798_G_1762_T_2463_A_2731 _(frequency 0.41) (underlined allele denotes change from the published Btau_4.0 sequence NC_007304.4). Owing to the low number of individuals typed for all five markers (*n *= 125), haplotype association analysis was not performed.

**Table 2:** Genotype and allele frequencies including significance from Hardy-Weinberg equilibrium for bo*TLR1 *SNPs selected for trait association.

SNP	Genotype	n =	Genotype Frequency	HWE	Allele	Allele Frequency
-79 T > G	TT	53	0.34	0.831	T	0.568

	TG	71	0.46		G	0.432

	GG	32	0.20			

+798 C > T^1^	CC	78	0.32	0.885	C	0.543

	CT	122	0.49		T	0.456

	TT	46	0.19			

+1762A > G^1^	AA	33	0.26	0.326	A	0.495

	GA	57	0.46		G	0.505

	GG	35	0.28			

+2463 C > T	CC	64	0.28	0.163	C	0.507

	CT	102	0.46		T	0.493

	TT	59	0.26			

+2731A > G	AA	77	0.33	0.611	A	0.546

	AG	110	0.48		G	0.454

	GG	45	0.19			

^1^SNPs genotyped by RFLP-PCR. HWE not significant at 1 degree of freedom.

### Analysis of bo*TLR1 *SNPs with clinical mastitis

The three bo*TLR1 *SNPs -79 T > G, +1762A > G and +2463 C > T were analysed in Holstein Friesian cows for association with CM and milk production traits. Analysis of the tagging SNP -79 T > G revealed the TG and GG genotypes associated with an increased rate of CM (0.75 and 0.70 cases/cow/year, respectively) compared with the TT genotype (0.40 cases/cow/year) (Table 3). The association with CM rate was found to be significantly (*P *< 0.05) higher for the heterozygote (TG) population, the most abundant genotype, when compared with homozygote (TT) animals. The proportion of animals within each genotyped population having any episodes of CM during their first three lactations was highest in the GG genotype (70%) compared with TT and TG genotypes (58% and 60%, respectively), although this was found not to be statistically significant. Analysis of the non-synonymous, transmembrane domain SNP; +1762A > G, showed AA and AG animals had higher rates of CM (0.69 and 0.66 cases/cow/year, respectively) compared with GG animals (0.38 cases/cow/year), although these differences were not significantly different. Analysis of the SNP +2463 C > T located within the 3'UTR revealed that CC and CT animals had higher rates of CM (0.70 cases/cow/year) compared to TT animals (0.39 cases/cow/year). However, only the increase seen in the most abundant genotype, heterozygous CT cows, was shown to be statistically significant (*P *< 0.05).

**Table 3:** Analysis of bo*TLR1 *SNP genotypes by CM and milk productivity traits over the first three lactations.

SNP	**Gen**.	n =	P% with CM^1^	Mean CM rate^2^	*P = *^3^	Median SCC^4^	*P = *^3^	Mean 305 d milk yield^4^	*P = *^3^	Mean milk fat (%)^4^	*P = *^3^	Mean milk protein (%)^4^	*P = ^3^*
tag-79 T > G	TT	78*	58%	0.40 (± 0.11)		1.96 (± 0.63)		7868 (± 1181)		4.15 (± 0.41)		3.28 (± 0.19)	

	TG	122*	60%	0.75 (± 0.18)	< 0.05	1.85 (± 0.61)	-	8058 (± 1139)	-	4.02 (± 0.38)	< 0.05	3.2 (± 0.17)	< 0.01

	GG	46*	70%	0.70 (± 0.24)	-	1.98 (± 0.67)	-	7983 (± 1273)	-	3.89 (± 0.41)	< 0.01	3.16 (± 0.18)	< 0.001

+1762A > G	AA	33	63%	0.69 (± 0.27)	-	1.96 (± 0.64)	-	8101 (± 1106)	-	3.95 (± 0.41)	-	3.19 (± 0.18)	-

	GA	57	61%	0.66 (± 0.18)	-	1.89 (± 0.69)	-	8067 (± 1330)	-	4.04 (± 0.45)	-	3.21 (± 0.2)	-

	GG	35	59%	0.38 (± 0.16)		1.90 (± 0.62)		8239 (± 1185)		4.05 (± 0.38)		3.25 (± 0.16)	

+2463 C > T	CC	64	67%	0.70 (± 0.24)	-	1.95 (± 0.66)	-	7841 (± 1067)	-	4.02 (± 0.45)	-	3.19 (± 0.18)	< 0.05

	CT	102	61%	0.70 (± 0.14)	< 0.05	1.90 (± 0.63)	-	8137 (± 1206)	-	3.98 (± 0.45)	-	3.18 (± 0.2)	< 0.05

	TT	59	59%	0.39 (± 0.11)		1.88 (± 0.63)		7940 (± 1262)		4.13 (± 0.38)		3.27 (± 0.18)	

Abbreviations: Gen. = genotype, P% = population percentage, CM = clinical mastitis, SCC = somatic cell count. * Numbers from tagged SNP +798 C > T in Table 2. ^1^No significance was detected between genotypes by Chi^2 odds ratio test. ^2^Mean ± 95% confidence interval given in column parentheses. ^3^*P *values analysed by ANOVA and Tukey's multiple comparisons, showing only results tested against the underlined (lower CM rate) genotype; - (dash) denotes no significance. ^4^Standard deviation from the mean given in column parentheses.

### Association of bo*TLR1 *SNPs with milk productivity traits

Analysis of the productivity traits within the genotyped animals revealed that the TG and GG genotypes of the tagging SNP -79 T > G associated significantly with lowered milk fat (TG = *P *< 0.05, GG = *P *< 0.01) and protein (TG = *P *< 0.01, GG = *P *< 0.001) concentrations when compared to those observed in the TT genotype (Table 3). A similar trend was detected in the genotypes of the SNP +1762A > G; AA and AG genotypes associated with lower milk fat and protein concentrations; however these differences were not statistically significant. Analysis of genotypes within the SNP +2463 C > T revealed that CT and CC genotypes produced milk with lower protein concentration (*P *< 0.05) when compared with the TT population. The same genotypes tended to show lower milk fat concentration, but these differences were not significant. No differences in either milk yield or mean SCC were detected between any of the genotyped populations, for any of the SNPs described.

### Multiple comparisons

Correlation coefficient analysis was performed revealing a weak positive correlation between CM and SCC (Pearson coefficient = 0.198) and weak negative correlations between CM and milk fat and milk protein percentages (Pearson coefficient = -0.2 and -0.1 respectively). All significant associations between individual SNP genotypes and CM were found to survive adjustments for each factor using a General linear model (GLM).

### Q-PCR analysis of basal bo*TLR1 *mRNA abundance

**Figure 3:**
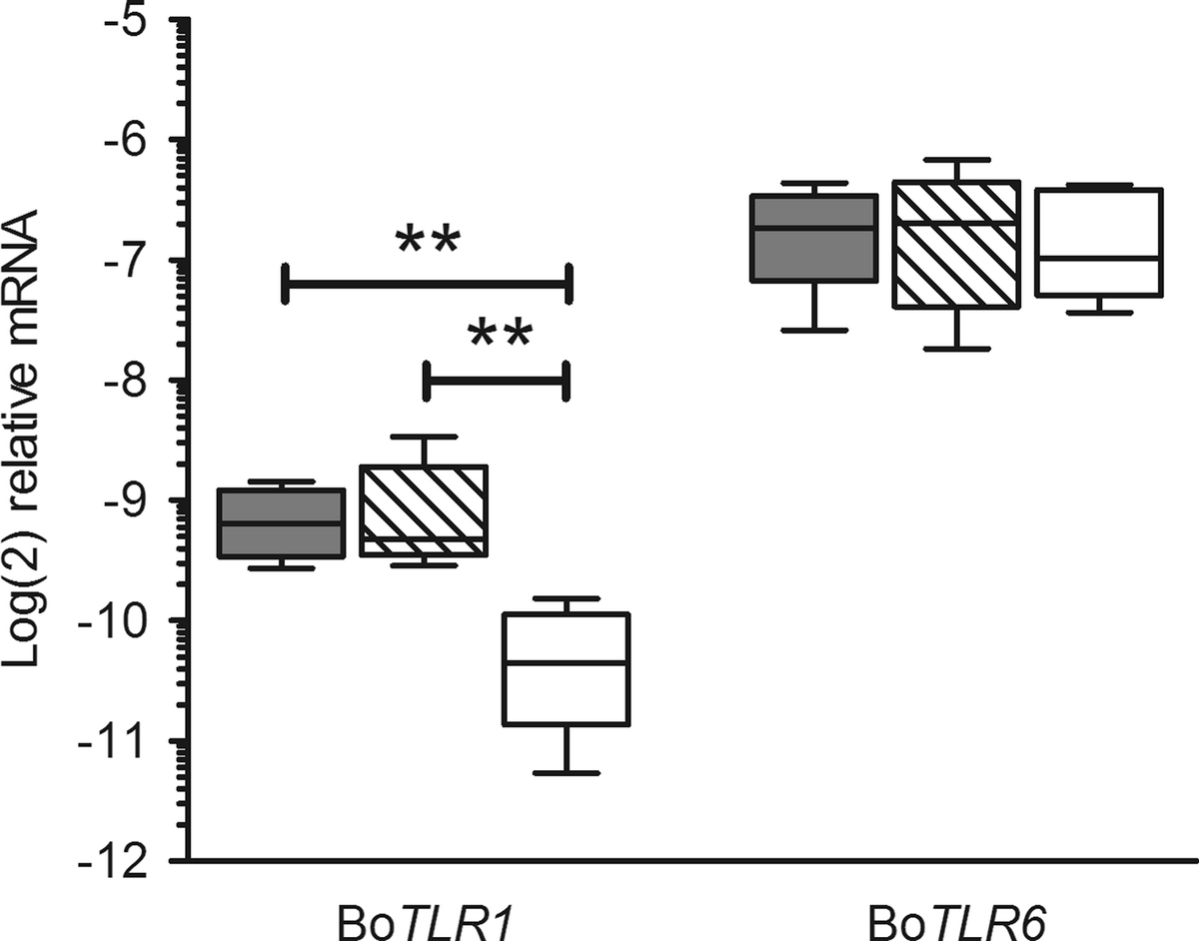
**Q-PCR data showing basal mRNA abundance levels of bo*TLR1 *and bo*TLR6 *in milk somatic cells grouped into the three bo*TLR1 *-79 T > G SNP variant populations: TT, TG and GG**. Data from each variant population (*n *= 5) is presented as a box and whisker plot, with the ends of the whiskers representing the minimum and maximum data values. Grey box plot - TT animals, hatched box plot - TG animals, and open box plot - GG animals. ** Genotypic means differ significantly (*P *< 0.01 by ANOVA and Tukey's multiple comparisons).

Milk somatic cells from cows with representative bo*TLR1 *-79 T > G SNP genotypes were assessed for differences in basal bo*TLR1 *expression. All animals were of similar age and stage of lactation and none had any known health issues. Q-PCR assays were performed using RNA from five animals of each genotype. Cows with the GG genotype demonstrated lower bo*TLR1 *mRNA abundance (*P *< 0.01) compared with either the TT or TG genotypes (Figure 3). Due to close homology and potential for co-expression, the abundance of bo*TLR6 *mRNA was also measured and revealed no significant differences.

### Variation in immune responses to a TLR1 ligand

**Figure 4:**
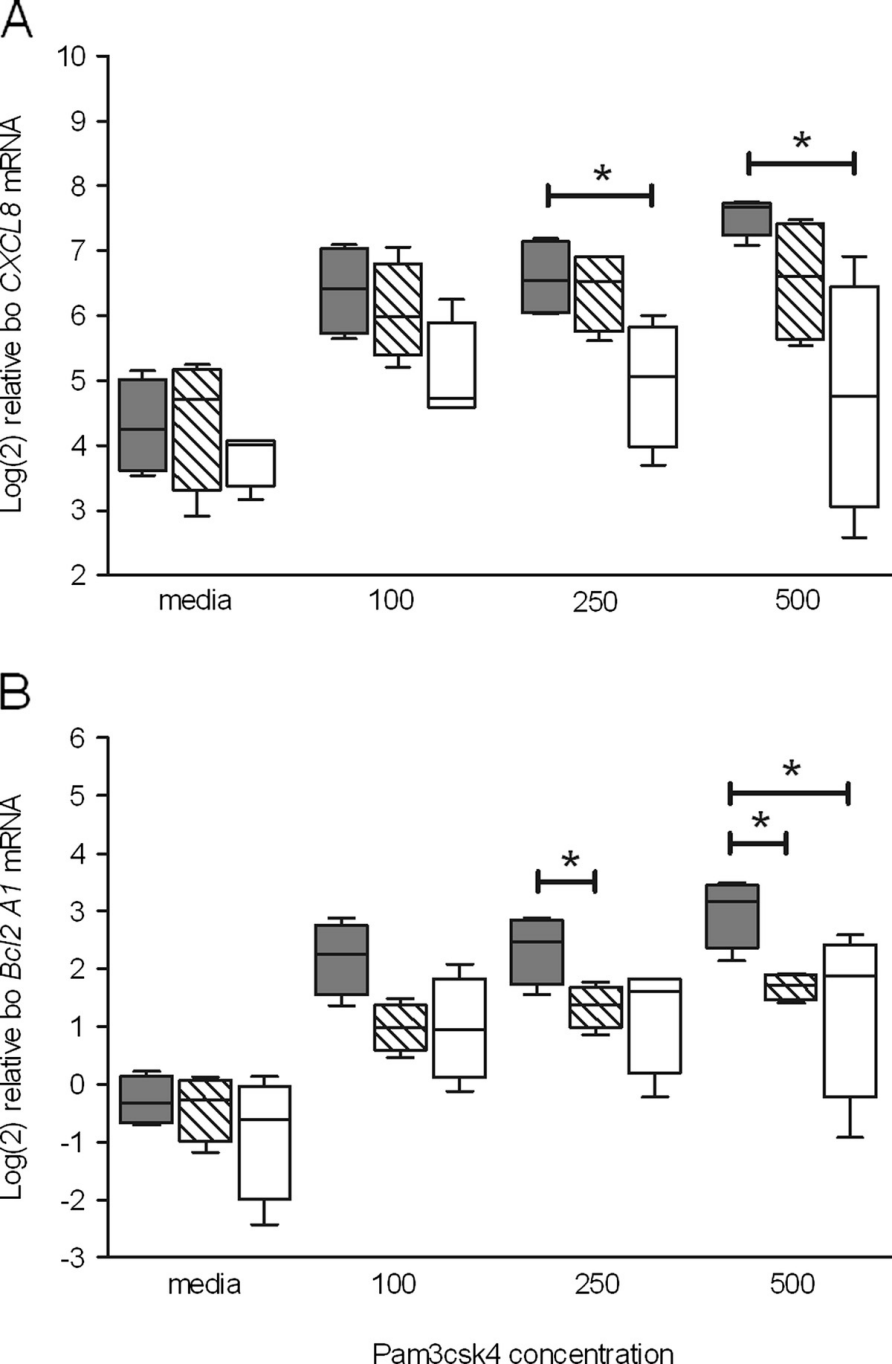
**Q-PCR data following stimulation with the TLR1 ligand PAM_3 _in bovine PMNs grouped into the three bo*TLR1 *-79 T > G SNP variant populations: TT, TG and GG. Q-PCR data of *CXCL8 *(A) and *Bcl2A1 *(B) mRNA abundance following +4 h stimulation**. Data from each variant population (*n *= 4) is presented as a box and whisker plot, with the ends of the whiskers representing the minimum and maximum data values. Grey box plot - TT animals, hatched box plot - TG animals, and open box plot - GG animals. *Genotypic means differ significantly (*P *< 0.05 by ANOVA and Tukey's multiple comparisons).

Cells from twelve animals (four from each representative bo*TLR1 *-79 T > G SNP genotype) were stimulated for up to 24 h with the ligand Pam3csk4 (PAM_3_), which has an affinity for TLR1/TLR2 heterodimers [49]. *CXCL8 *and *IL-6 *mRNA abundance and CXCL8 and IL-6 production were used as markers for TLR1 stimulation. Q-PCR analysis indicated that animals with the GG genotype had lower levels of *CXCL8 *mRNA 4 h post-stimulation with PAM_3 _compared to equivalent samples from animals with either of the TT or TG genotypes (Figure 4). Samples from animals that were homozygous TT showed significantly greater (*P *< 0.05) *CXCL8 *mRNA abundance 4 h after stimulation with PAM_3 _at concentrations of 250 and 500 ng/mL when compared to GG homozygotes (Figure 4a). Homozygous TT animals tended to show greater *IL-6 *abundance 4 h after stimulation however no significant differences were detected between genotypes across all ligand concentrations (data not shown). A similar trend in *CXCL8 *and *IL-6 *data was observed from cells stimulated for 8 h (data not shown). Animals that were homozygous TT displayed significantly greater (*P *< 0.05) *IL-6 *mRNA abundance 8 h after stimulation with PAM_3 _at 500 ng/mL when compared to GG homozygotes (data not shown).

The mRNA abundance of the anti-apoptotic factor *Bcl2A1*was used as an additional marker to indicate differences in TLR1 stimulation. Following stimulation with PAM_3_, Q-PCR analysis showed greater levels of *Bcl2A1 *mRNA in animals with the TT genotype compared to those with either the TG or GG genotypes (Figure 4b). Significantly (*P *< 0.05) greater levels of mRNA were detected in samples from animals with the TT genotype compared to those with the TG genotype following stimulation with PAM_3 _at 250 ng/mL and 500 ng/mL. In contrast, a statistically significant difference between the TT and GG genotypes was only detected following stimulation at the higher concentration.

**Figure 5:**
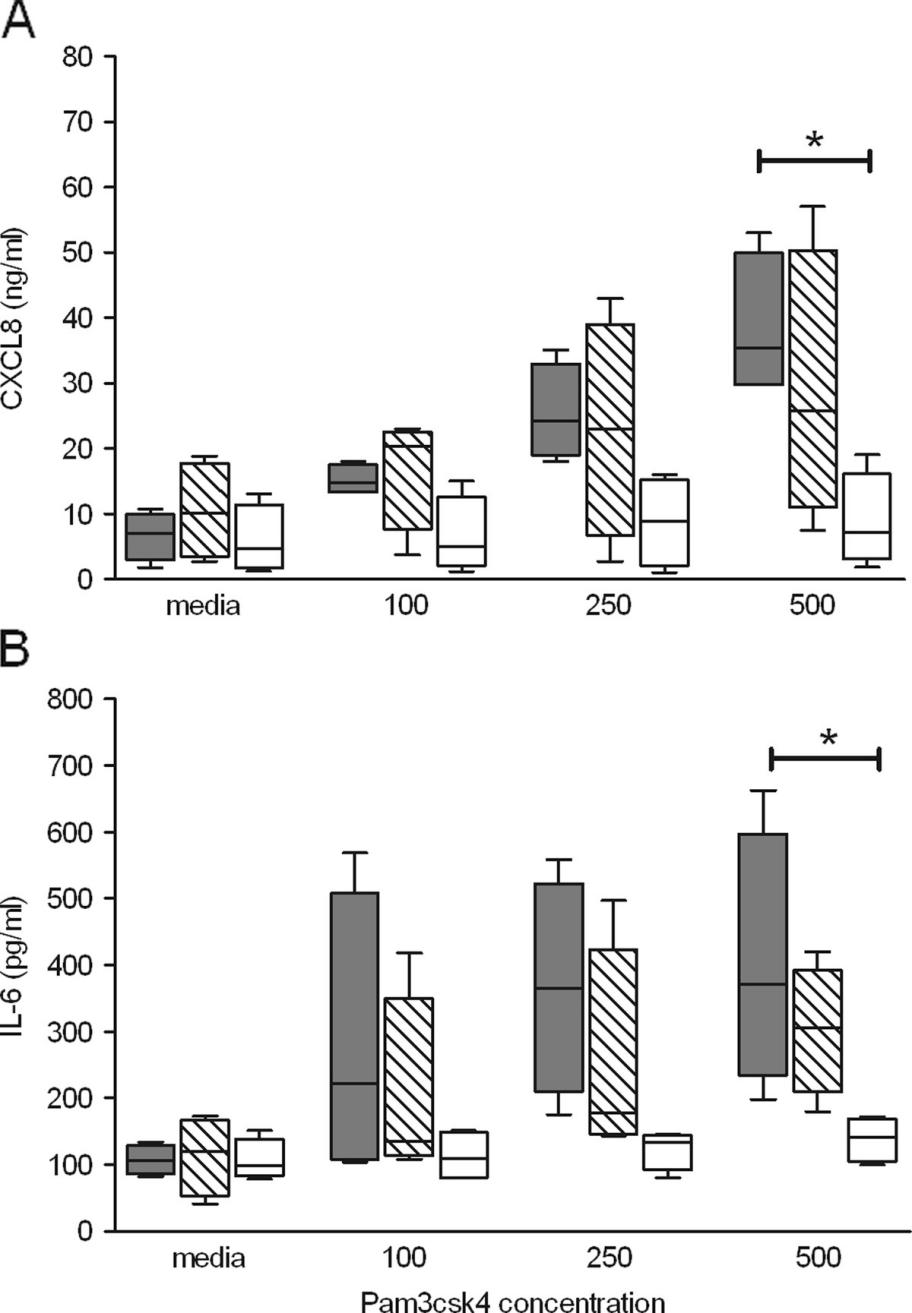
**ELISA data following stimulation with the TLR1 ligand PAM_3 _in bovine PMNs grouped into the three bo*TLR1 *-79 T > G SNP variant populations: TT, TG and GG. ELISA data of CXCL8 (A) and IL-6 (B) production following + 24 h stimulation**. Data from each variant population (*n *= 4) is presented as a box and whisker plot, with the ends of the whiskers representing the minimum and maximum data values. Grey box plot - TT animals, hatched box plot - TG animals, and open box plot - GG animals. * Genotypic means differ significantly (*P *< 0.05 by ANOVA and Tukey's multiple comparisons).

Supernatants (24 h post-stimulation) were assayed for CXCL8 and IL-6 production by ELISA. CXCL8 data showed a similar trend to those obtained by Q-PCR, with significantly (*P *< 0.05) lower levels of CXCL8 produced by cows with the GG genotype than those with the TT genotype following stimulation with 500 ng/mL PAM_3 _(Figure 5a). Individual variation was largest within those samples from TG heterozygotes, with higher concentrations of PAM_3 _resulting in increased variability in CXCL8 production. IL-6 data showed significantly (*P *< 0.05) lower levels produced by cows with the GG genotype than the TT genotype following stimulation with 500 ng/mL PAM_3 _(Figure 5b). Overall stimulation with TLR1 ligand across all concentrations did not increase cytokine production in GG variants. The TLR4 ligand LPS was used to assess if the detected variation in response to PAM_3 _reflected the ability of the cell populations to respond to any ligand. The response to LPS was variable across all genotypes and did not segregate by the defined genotypes (Additional file 2).

## Discussion

In this study we analysed five SNPs within exonic and non-exonic regions of bo*TLR1 *for susceptibility to CM. We identified significant associations between the tagging SNP -79 T > G, and the 3'UTR SNP +2463 C > T and susceptibility to CM. Rates of CM for genotypes -79 TT and +2463 TT were much lower in comparison to the homozygous genotypes (-79 GG and +2463 CC) and significantly lower than their respective heterozygous genotypes. The CM rate differences observed between defined genotypes can have a significant impact on both herd welfare and farm expenditure. For example, the -79 TT animals recorded on average 40 CM cases per 100 cows per year, below the historic national average for England and Wales, which has been estimated at 47 cases [1]. GG and TG animals recorded 70 and 75 cases respectively, a substantial increase from the national average. As a simple economic evaluation, if estimated average costs for a clinical case are £175 [50] potential losses per 100 cows when compared to TT animals equate to £6125 and £5250 for TG and GG animals respectively.

Dairy cows are subjected to intense selection pressures for higher milk yields and reduced SCC that may unintentionally be detrimental to CM incidence [5]. However, in this study, no significant differences were observed between bo*TLR1 *SNPs for mean 305 d milk yields or SCC. This contradicts a study of 208 Chinese Holsteins, in which the non-synonymous SNP +1762A > G associated with highly significant differences in milk SCC. Animals with an AA (Isoleucine) at this position had lower SCC and were classed as more resistant to mastitis [36]. In contrast, our results suggest the GG (Valine) at this location is the more favourable genotype in terms of having a lower CM incidence in comparison to AA. Although this discrepancy may reflect breed and herd variation, it may also demonstrate the dichotomy that the surrogate measure of mastitis is also the presence of the effector cell which can at times eliminate infection in the absence of overt clinical signs.

All bo*TLR1 *SNP genotypes associated with a lowered rate of CM display significantly greater milk fat and protein concentrations when compared to the other two genotypes. It is unclear whether a functional association exists between milk fat and protein concentrations and mastitis susceptibility. Interestingly the casein gene cluster is located ~28 Mb from the *TLR6-TLR1-TLR10 *gene cluster on bta6 [51]. Protein and fat contents are financially rewarded milk qualities and breeding for these factors could influence bo*TLR1 *genotypes.

In addition to being one of the first studies to examine association between bo*TLR1 *SNPs and CM incidence, we further demonstrate evidence of variable receptor responses between the defined genotypes. In this study animals segregated by -79 T > G SNP genotypes exhibited significantly lower bo*TLR1 *expression in GG animals when compared with TT or TG. This difference, which appeared to be specific for bo*TLR1 *as it was not seen for bo*TLR6 *a paralogue of bo*TLR1*, may indicate interference with transcription. Although more variable, intermediate basal expression levels observed in TG cows suggests that the presence of a T allele is compensating for the lowered expression originating from the G allele. Preliminary analysis for causal mutations using the programme MOTIF [52] highlighted the -79 T > G SNP as part of an Octamer-1 (Oct-1) motif. Oct-1 is thought to work closely with AP-1 and GATA-1 as important regulatory elements for TLR expression [53]. However the motif was only identified with the T variant (threshold > 80%), and was absent in those with the G substitution. Although this is an attractive hypothesis it is also possible that the SNP may simply be a marker for other unidentified causal mutations.

Currently there are no boTLR1 antibodies available to directly compare levels of protein expression. To ascertain the functional consequence of potentially reduced bo*TLR1 *expression, production of cytokines known to be induced by TLR1 was determined from cells stimulated with the TLR1 ligand PAM_3_. Our study demonstrated genotype-specific variation in cytokine responses of bovine PMN to PAM_3_. Across all time points and ligand concentrations analysed, PAM_3 _stimulation of cells from animals segregated by tagging -79 T > G SNP genotypes revealed lower levels of CXCL8 and IL-6 mRNA and protein from those of a GG genotype and consistently high CXCL8 and IL-6 response from those with TT genotypes. The output of cytokines from cells from the TG animals was the most variable. The NFκB-transcribed gene [54] encoding the pro-survival protein *Bcl2A1*, which is up-regulated in neutrophils following stimulation with TLR ligands including PAM_3 _[55], was used as an additional marker of effective bo*TLR1 *stimulation. Significantly greater levels of the *Bcl2A1 *transcript were detected in animals with the TT genotype compared to those with the TG or GG genotypes. All genotypes were also stimulated with LPS, and no differences were detected in levels of CXCL8 or IL6. This further indicates that differences observed following stimulation with PAM_3 _were influenced by variation in the response via bo*TLR1*.

PMNs contain a high proportion of neutrophils, whose primary role is the phagocytosis of microbes to contain and eliminate infection. However stimulated neutrophil TLRs further induce the neutrophil to synthesise cytokines that influence their own activity and survival, recruit additional immune cells and modulate the immune response [56-59]. The greater expression of *Bcl2A1 *in animals with the TT genotype may promote PMN survival to complement the increased cytokine output. Reduced boTLR1 stimulation by pathogens in GG cows may equate to a dampened inflammatory response thus enabling establishment and persistence of infection. In terms of influence upon mastitis susceptibility, GG cows have a higher CM rate than TT cows; however heterozygous TG cows have a similar rate to GG cows, despite expressing bo*TLR1 *at a similar level to a TT cow. TG animals showed intermediary cytokine responses to TLR1 ligand, and like GG animals, lower expression of *Bcl2A1*. This may suggest that differences in bo*TLR1 *transcript levels alone may not account for the variation in receptor response, and/or that post-transcriptional control of translation may also be affected by the cumulative presence of SNPs. The animals used for the functional assays conform to observed haplotypes: TT = T_79_C_798_G_1762_T_2463_A_2731 _and GG = G_79_T_798_A_1762_C_2463_G_2731_; with TG animals heterozygous for each SNP. In terms of the likelihood of being causative of a variable TLR1 response, the non-synonymous SNP +1762 Iso > Val is an unlikely candidate. Both Isoleucine and Valine are non-polar branched amino acids with hydrophobic properties, and so a substitution within the transmembrane domain should not impact the tertiary structure; although further targeted structure/function studies are required to verify this. Furthermore, influence of polymorphisms within TLR-related or target genes such as *TLR2 *and *CXCL8 *should be considered.

In conclusion the findings presented here demonstrate an association between SNPs, common to bo*TLR1*, and CM susceptibility. Furthermore, we present evidence that some of these SNPs underpin functional variation in boTLR1. A rapid immune response, conferred by the favourable bo*TLR1 *SNP -79 TT variant, could reduce detrimental clinical manifestations of diseases via an efficient yet controlled influx of inflammatory cells to neutralise pathogens and control infection. The complexity of mastitis infection suggests a polygenic and multi-factorial immune response comprising many different proteins which will in turn interact with one another. The ability to detect significant associations with CM for a single gene indicates the importance of boTLR1 related responses in the control of bacterial disease in cattle. A bovine population with impaired bo*TLR1 *expression may also be more susceptible to other diseases and health issues. Preliminary evidence for this comes from our study where it was observed that animals with the GG variant seemed to be prematurely culled compared to other populations.

The potential of SNPs within bo*TLR1 *to act as genetic markers for altered susceptibility to mastitis warrants further investigation. The favourable linkage of lowered CM with increased milk fat and protein concentrations in the bo*TLR1 *variants demonstrated here indicates selection for lowered CM using these markers would not be detrimental to milk quality, as has been previously suggested in studies linking SNPs with increased SCC as a marker for CM susceptibility.

## Competing interests

The authors declare that they have no competing interests.

## Authors' contributions

Conceived and designed the experiments: CDR, SW, JAL, TJC. Performed the experiments: CDR. Analysis and discussion of data: CDR, SW, JAL, TJC. Wrote the manuscript: CDR. Edited the manuscript: SW, JAL, TJC. All authors read and approved the final manuscript.

## Supplementary Material

Additional file 1 **Primers used for PCR amplification of bo*TLR1 *genomic regions, RFLP PCR digests, and quantitative PCR (QPCR) assays**. Click here for file

Additional file 2 **Table to show expression levels of *CXCL8 *and *Bcl2 A1 *and CXCL8 and IL-6 production levels following PMN stimulation with LPS**. Click here for file
