# Supplementary material for: Identification of single nucleotide polymorphisms in the bovine Toll-like receptor 1 gene and association with health traits in cattle
Source: Vet Res. 2012 Mar 14;43(1):17. doi: 10.1186/1297-9716-43-17 (PMC3342155; doi:10.1186/1297-9716-43-17)
Supplement: Additional file 1 — Primers used for PCR amplification of boTLR1 genomic regions, RFLP PCR digests, and quantitative PCR (QPCR) assays. [file 1297-9716-43-17-S1.PDF]

Additional File 1 Primers used for PCR amplification of bo*TLR1* genomic regions, RFLP PCR digests, and quantitative PCR (QPCR) assays.

**Bo*TLR1* Region**	**Annealing Temp (°C)**	**Sequence (5'- 3')**	**Product size**
5' Upstream region (UTR intronic)	59	Forward - GCCTCCTTACTGTGCCTGAG<br>Reverse - TGGTTGTTTCCAGGGATAAGTT	1910bp
5' UTR region to coding +320bp	58	Forward - GGACATGGTTAGGAGGTGGA<br>Reverse - TTGTTGTGGGACAAATCCAA	777bp
5' UTR region to coding +820bp	59	Forward - GGACATGGTTAGGAGGTGGA<br>Reverse - GTACGCCAAACCAACTGGAG	1278bp
Coding +650-1760bp	59	Forward - GCACCACAGTGAGTCTGGAA<br>Reverse - TTCTTCACCCAGGCAGAATC	1327bp
Coding +1671- 3' UTR region	60	Forward - AGGGCTGGCCTGAGTCTTAT<br>Reverse - CTGGGGCCTGAAAAGACATA	1240bp
Coding +2160 -3' UTR region	58	Forward - ACTTTGCCCACCACAATCTC<br>Reverse - CTGGGGCCTGAAAAGACATA	748bp
SNP C+798T Mbo II digest	59	Forward - GCACCACAGTGAGTCTGGAA<br>Reverse - GTACGCCAAACCAACTGGAG	181bp
SNP A+1762G Bcl I digest	60	Forward - AGGGCTGGCCTGAGTCTTAT<br>Reverse - TTCTTCACCCAGGCAGAATC	316bp
RPLP2 QPCR	58	Forward - TCAACAAGGTCATCAGTGAGC<br>Reverse - CCGATACCCTGAGCAATGA<br>Probe - CGTCCTCGATGTTCTTTCCGTGG	64bp
TLR1 QPCR	59	Forward - GCACCACAGTGAGTCTGGAA<br>Reverse - GTACGCCAAACCAACTGGAG<br>Probe - TGTGTGCTTGATGATAATGGGTGTCCT	181bp
TLR6 QPCR	59	Forward - CCTGCCCATCTGTAAGGAAT<br>Reverse - TAGGTGCAAGTGAGCAATGG<br>Probe - TTGGCAACTTGACCCAACTGAATTTC	109bp
