# Supplementary material for: Identification of single nucleotide polymorphisms in the bovine Toll-like receptor 1 gene and association with health traits in cattle
Source: Vet Res. 2012 Mar 14;43(1):17. doi: 10.1186/1297-9716-43-17 (PMC3342155; doi:10.1186/1297-9716-43-17)
Supplement: Additional file 2 — Table to show expression levels of CXCL8 and Bcl2 A1 and CXCL8 and IL-6 production levels following PMN stimulation with LPS. [file 1297-9716-43-17-S2.PDF]

Additional File 2 Expression levels of *CXCL8* and *Bcl2 A1* and CXCL8 and IL-6 production levels following PMN stimulation with LPS.

	bo*TLR1* -79T>G SNP genotype		
	TT	TG	GG
CXCL8	75 (48-126)	135 (47-186)	94 (34-160)
Bcl2 A1	4.4 (3.4-6.9)	4.4 (3.7-7.4)	5 (2.5-8.9)
CXCL8 (ng/µl)	24 (19-50)	50 (20-59)	38 (31-50)
IL-6 (pg/µl)	390 (220-656)	382 (167-407)	420 (346-462)

Median relative expression levels of *CXCL8* and *Bcl2 A1* transcripts (+4h) and median CXCL8 and IL-6 production levels (+24h) following PMN stimulation with the TLR4 ligand LPS from animal samples segregated by tagging bo*TLR1* SNP -79T>G genotypes: TT, TG and GG. Minimum and maximum values are given in parenthesis (min-max) to allow comparison to box plots in Figure 4 and Figure 5. No significant differences ($P < 0.05$) were observed between genotype means using One-way ANOVA and Tukey's multiple comparison tests.
